# Palladium-catalyzed 2,5-diheteroarylation of 2,5-dibromothiophene derivatives

**DOI:** 10.3762/bjoc.10.309

**Published:** 2014-12-09

**Authors:** Fatma Belkessam, Aidene Mohand, Jean-François Soulé, Abdelhamid Elias, Henri Doucet

**Affiliations:** 1 Institut des Sciences Chimiques de Rennes, UMR 6226 CNRS-Université de Rennes, "Organométalliques: Matériaux et Catalyse", Campus de Beaulieu, 35042 Rennes, France, Tel.: 00-33-2-23-23-63-84, Fax 00-33-2-23-23-69-39; 2 Département de chimie, Tizi Ouzou University, BP 17 RP 15000 Tizi-Ouzou, Algeria

**Keywords:** aryl halides, catalysis, C–H bond activation, direct arylation, heteroarenes, palladium

## Abstract

Conditions allowing the one pot 2,5-diheteroarylation of 2,5-dibromothiophene derivatives in the presence of palladium catalysts are reported. Using KOAc as the base, DMA as the solvent and only 0.5–2 mol % palladium catalysts, the target 2,5-diheteroarylated thiophenes were obtained in moderate to good yields and with a wide variety of heteroarenes such as thiazoles, thiophenes, furans, pyrroles, pyrazoles or isoxazoles. Moreover, sequential heteroarylation reactions allow the access to 2,5-diheteroarylated thiophenes bearing two different heteroaryl units.

## Introduction

**Figure 1:**
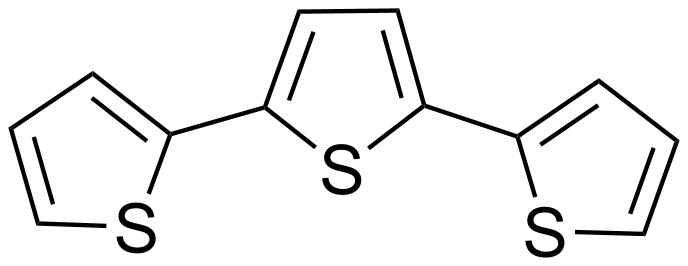
2,2':5',2"-Terthiophene.

2,2':5',2"-Terthiophene (or 2,5-di(2-thienyl)thiophene) (Figure 1) and many of its derivatives are important structures due to their biological and/or physical properties. For example, 2,2':5',2"-terthiophene itself is a pigment of Tagetes minuta. Some 2,2':5',2"-terthiophene derivatives such as 5,5''-dichloro-α-terthiophene also occur naturally [1]. Moreover, terthiophenes are widely used as building blocks for the synthesis of semiconductors [2]. Due to these multiple uses, the discovery of a simpler access to terthiophene derivatives would be very useful.

Suzuki, Stille or Negishi Pd-catalyzed cross-coupling reactions represent some of the most efficient methods for the preparation of 2,5-diheteroarylated thiophenes [3–16]. However, an organometallic derivative must be prepared to perform such reactions. In 1990, Ohta and co-workers reported the Pd-catalyzed direct arylation of heteroaromatics using aryl halides as coupling partners via a C–H bond activation [17–18]. Since then Pd-catalyzed direct arylation of heteroaryls, especially with aryl halides as coupling partners, has been shown to be a very powerful method for an easier and greener access to a very broad range of arylated heterocycles [19–32]. This method is more attractive than other Pd-catalyzed cross-coupling reactions as it avoids the preparation of an organometallic derivative and also as the major byproducts of the reaction are not metallic salts but a base associated to HX.

The metal-catalyzed direct arylation of a wide variety of heteroarenes using aryl halides as coupling partners has been reported in recent years [19–36]. However, to our knowledge, only a few examples of Pd-catalyzed direct arylations at both C2 and C5 carbons of 2,5-dihalothiophene derivatives have been described. In 2006, Borgese et al. reported the Pd-catalyzed coupling of 2,5-dibromothiophene with 3-methoxythiophene to afford the corresponding terthiophene in 29% yield [37]. From 2,5-diiodothiophene and benzoxazole, using 5 mol % Pd(phen)_2_(PF_6_)_2_ catalyst, the 2,5-diheteroarylated thiophene was obtained in 89% yield by Murai et al. [38]. A fluorescent π-conjugate thiophene derivative bearing spiro[fluorene-9,4’-[4*H*]indeno[1,2-*b*]furan] substituents at C2 and C5 has been prepared in 46% yield by this reaction using Pd(OAc)_2_ (5 mol %) associated to PPh_3_ (10 mol %) as catalytic system [39]. A pyrrole derivative was coupled with 2,5-dibromothiophene in the presence of Pd(OAc)_2_ (5 mol %) and PCy_3_ (10 mol %) catalyst to afford the 2,5-di(pyrrolyl)thiophene in 59% yield [40]. Finally, an indolizine was also successfully coupled with 2,5-dibromothiophene in 47% yield in the presence of Pd(OAc)_2_ as catalyst [41]. To our knowledge, so far sequential Pd-catalyzed direct couplings using 2,5-dihalothiophene derivatives have not been described. Therefore, the discovery of effective general conditions, for the direct coupling of heteroarenes at both C2 and C5 positions of 2,5-dihalothiophene derivatives, would constitute a considerable advantage allowing a simpler access to terthiophene derivatives.

Here, we wish to report (i) that only 0.5–2 mol % of air-stable palladium catalysts associated to KOAc promote the direct access to 2,5-diheteroarylated thiophenes in one pot, (ii) on the reaction scope using a large set of heteroarenes, and (iii) conditions allowing the sequential diheteroarylation of 2,5-dibromothiophene.

## Results and Discussion

**Scheme 1:**
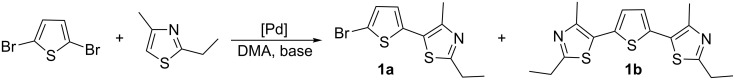
Palladium-catalyzed direct arylation using 2,5-dibromothiophene and 2-ethyl-4-methylthiazole as coupling partners.

Based on our previous results, DMA was initially chosen as the solvent and KOAc as the base for this study [42–43]. The reactions were conducted at 140 °C under inert conditions using PdCl(C_3_H_5_)(dppb) or Pd(OAc)_2_ catalysts. Using only 0.5 mol % Pd(OAc)_2_, the reaction of 1 equiv of 2,5-dibromothiophene with 2 equiv 2-ethyl-4-methylthiazole as coupling partners affords the mono- and diarylation products **1a** and **1b** in a 2:98 ratio and the desired product **1b** was isolated in 79% yield (Scheme 1, Table 1, entry 1). The use of 3 equiv of 2-ethyl-4-methylthiazole afforded **1b** in similar yield (Table 1, entry 2). Then, we examined the influence of the amount of catalyst and other parameters on the reaction. The use of 1 or 2 mol % PdCl(C_3_H_5_)(dppb) catalyst, which had been previously found to be very effective to promote the direct arylation of several hereroaromatics [42–44], also afforded **1b** in high yields (Table 1, entries 3–5). Even at 100 °C, the desired product **1b** was obtained in 78% yield (Table 1, entry 6). When CsOAc was employed as the base instead of KOAc, in the presence of 2 mol % PdCl(C_3_H_5_)(dppb) catalyst, **1b** was isolated in 80% yield, whereas NaOAc led to target product **1b** in only 68% yield and Cs_2_CO_3_ was ineffective (Table 1, entries 7–9). It should be noted that in the presence of an excess of 2,5-dibromothiophene (4 equiv) with 1 equiv of 2-ethyl-4-methylthiazole the products **1a** and **1b** were produced in a 72:28 ratio and **1a** was isolated in 52% yield, without cleavage of the second C–Br bond on the thiophene ring allowing sequential arylations (Table 1, entry 10).

**Table 1:** Influence of the reaction conditions for palladium-catalyzed direct arylation using 2,5-dibromothiophene and 2-ethyl-4-methylthiazole as coupling partners (Scheme 1).^a^

Entry	Catalyst (mol %)	Base	2-Ethyl-4-methylthiazole (equiv)	Temperature (°C)	Ratio **1a**:**1b**	Yield in **1b** (%)

1	Pd(OAc)_2_ (0.5)	KOAc	2	140	2:98	79
2	Pd(OAc)_2_ (0.5)	KOAc	3	140	1:99	80
3	PdCl(C_3_H_5_)(dppb) (2)	KOAc	3	140	0:100	81
4	PdCl(C_3_H_5_)(dppb) (1)	KOAc	3	140	0:100	80
5	PdCl(C_3_H_5_)(dppb) (2)	KOAc	2.2	140	1:99	78
6	PdCl(C_3_H_5_)(dppb) (2)	KOAc	3	100	0:100	78
7	PdCl(C_3_H_5_)(dppb) (2)	NaOAc	3	140	7:93	68
8	PdCl(C_3_H_5_)(dppb) (2)	CsOAc	3	140	0:100	80
9	PdCl(C_3_H_5_)(dppb) (2)	Cs_2_CO_3_	3	140	nd	<5
10	PdCl(C_3_H_5_)(dppb) (2)	KOAc	3	140	72:28	52^b^

^a^Conditions: 2,5-dibromothiophene (1 equiv), base (3 equiv), DMA, 20 h, isolated yields. ^b^2,5-Dibromothiophene (4 equiv), 2-ethyl-4-methylthiazole (1 equiv), yield in **1a**.

**Scheme 2:**
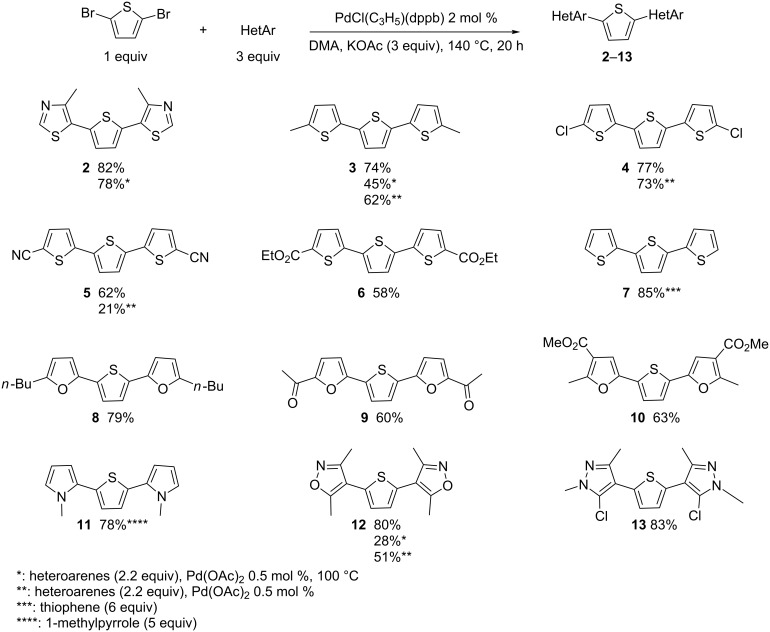
Reactivity of 2,5-dibromothiophene with different heteroarenes.

Then, with the most effective reaction conditions in hand for diheteroarylation (DMA, KOAc, Pd(OAc)_2_ or PdCl(C_3_H_5_)(dppb), 100 or 140 °C, 20 h), we explored the scope of this reaction using a variety of heteroarenes as the coupling partner (Scheme 2).

First, we investigated the reaction of 2,5-dibromothiophene with 4-methylthiazole (Scheme 2). The reaction proceeded very smoothly to afford the product **2** in 82% yield. It should be noted that no arylation at C2 of this thiazole derivative was observed. Then, a set of thiophene derivatives was employed. Both, 2-methyl- and 2-chlorothiophenes afforded the desired products **3** and **4** in good yields in the presence of PdCl(C_3_H_5_)(dppb) as the catalyst. Yields of 62% and 73% of these two products were obtained using 0.5 mol % Pd(OAc)_2_ catalyst at 140 °C, whereas a reaction performed at 100 °C led to only a partial conversion of 2,5-dibromothiophene to afford **3** in 45% yield. This slightly lower reactivity of thiophene derivatives under these conditions was expected, as they are known to be less reactive than thiazole derivatives [44]. Moderate yields for **5** and **6** were obtained starting form thiophene-2-carbonitrile and ethyl thiophene-2-carboxylate, respectively in the presence of 2 mol % PdCl(C_3_H_5_)(dppb) catalyst due to the formation of unidentified degradation products. The use of 6 equiv of thiophene allowed the formation of 2,2':5',2"-terthiophene (**7**) in 85% yield. The reactivity of three furan derivatives was also studied using PdCl(C_3_H_5_)(dppb) as the catalyst. From 2-*n*-butylfuran, **8** was obtained in 79% yield, whereas 2-acetylfuran and methyl 2-methylfuran-3-carboxylate afforded **9** and **10** in 60% and 63% yield, respectively. The reaction of 1 equiv of 2,5-dibromothiophene with 5 equiv of 1-methylpyrrole gave **11** in 78% yield. No significant formation of other polyheterocycles was observed by GC–MS analysis of the crude mixture. Arylation at C4 of 3,5-dimethylisoxazole and 5-chloro-1,3-dimethylpyrazole afforded **12** and **13** in 80% and 83% yields, respectively. With 3,5-dimethylisoxazole, a reaction performed using only 0.5 mol% Pd(OAc)_2_ catalyst at 100 °C led to a partial conversion of 2,5-dibromothiophene.

**Scheme 3:**
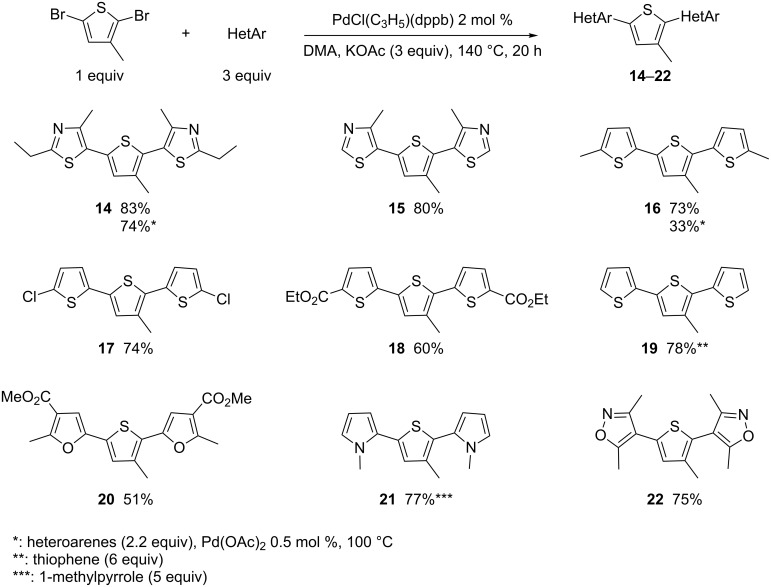
Reactivity of 2,5-dibromo-3-methylthiophene with different heteroarenes.

As several terthiophene derivatives bearing alkyl substituents at C3 in their central unit have been employed in material chemistry [2], the reactivity of 2,5-dibromo-3-methylthiophene was also examined (Scheme 3). Similar results to those of 2,5-dibromothiophene were obtained. Both, 2-ethyl-4-methylthiazole and 4-methylthiazole reacted nicely to afford **14** and **15** in 83% and 80% yields, respectively. The four terthiophenes **16**–**19** were also obtained in satisfactory yields. Again a moderate yield in **20** was obtained in the presence of methyl 2-methylfuran-3-carboxylate due to the formation of degradation products, whereas the reaction with 1-methylpyrrole and 3,5-dimethylisoxazole resulted in good yields of **21** and **22**, respectively.

**Scheme 4:**
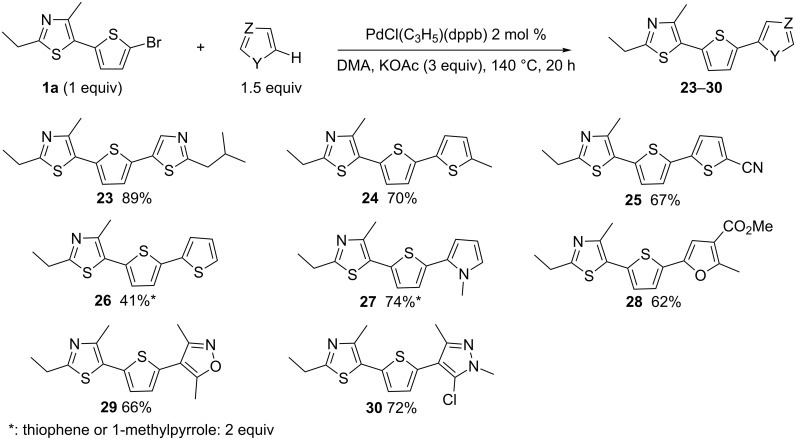
Sequential diheteroarylation of 2,5-dibromothiophene.

To our knowledge, the sequential Pd-catalyzed direct diheteroarylation of 2,5-dibromothiophene has not yet been reported. A sequential heteroarylation would allow the synthesis of non-symmetrically 2,5-disubstituted thiophene derivatives. Our attempts to prepare these compounds are shown in Scheme 4. Eight heteroarenes were reacted with **1a** to afford the 2,5-diheteroarylated thiophenes **23**–**30** in 41–89% yield. A high yield of 89% for **23** was obtained from **1a** and 2-isobutylthiazole as the coupling partners. The reactions with 2-methylthiophene and thiophene-2-carbonitrile also afforded the desired products **24** and **25** in good yields. A decreased yield of 41% for **26** was obtained with thiophene as coupling partner, whereas, 1-methylpyrrole gave **27** in 74%. Coupling of **1a** with methyl 2-methylfuran-3-carboxylate afforded **28** in 62% yield. The arylation at C4 of 3,5-dimethylisoxazole and 5-chloro-1,3-dimethylpyrazole also proceeded nicely to give **29** and **30** in 66% and 72% yield, respectively.

**Scheme 5:**
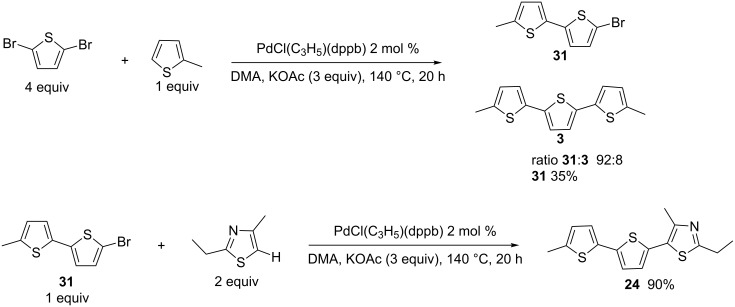
Sequential diheteroarylation of 2,5-dibromothiophene.

It should be noted that, for the synthesis of **24**, the introduction of the thiazole unit in the first step (Scheme 1 and Scheme 4, 36% over 2 steps) led to a slightly higher yield than the introduction of 2-methylthiophene followed by the coupling with 2-ethyl-4-methylthiazole (Scheme 5, 32% yield over 2 steps).

**Scheme 6:**
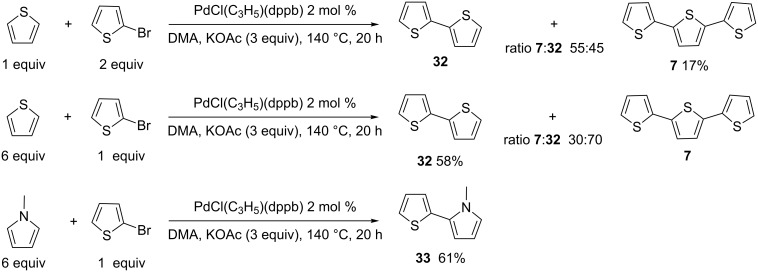
Heteroarylation of 2-bromothiophene.

We also compared the preparation of 2,2':5',2"-terthiophene (**7**) starting from either 2,5-dibromothiophene (Scheme 2) or from 2-bromothiophene (Scheme 6). The reaction of 1 equiv thiophene with 2 equiv of 2-bromothiophene resulted in a poor yield for **7** due to the formation of a mixture of bithiophene **32**, terthiophene **7** and also a quaterthiophene (as was observed by GC–MS analysis of the crude mixture). On the other hand, the use of 6 equiv of thiophene in the presence of 1 equiv of 2-bromothiophene afforded **7** and **32** in a 30:70 ratio and only low amounts of a quaterthiophene were observed; compound **32** was isolated in 58% yield (Scheme 6, middle). The same reaction conditions allowed to prepare 1-methyl-2-(thiophen-2-yl)pyrrole (**33**) in 61% yield (Scheme 6, bottom).

**Scheme 7:**
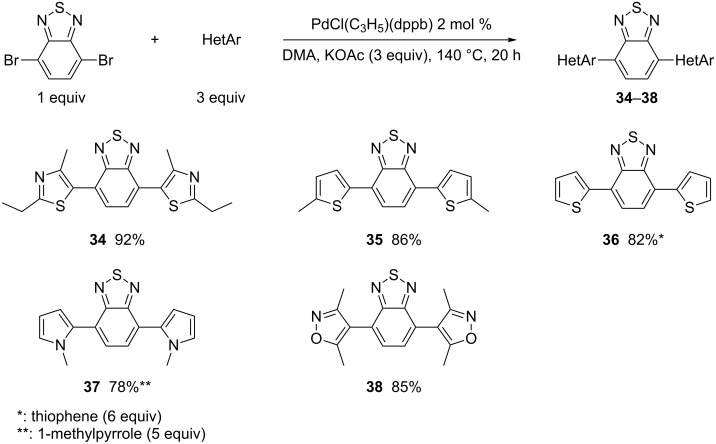
Reactivity of 4,7-dibromobenzothiadiazole.

Finally, as 4,7-diarylbenzothiadiazoles also display important physical properties [45], we applied our procedure to 4,7-dibromobenzothiadiazole which is commercially available (Scheme 7). In all cases, the desired 4,7-diarylbenzothiadiazoles **34**–**38** were obtained in high yields.

## Conclusion

In summary we report here a simple one-pot catalytic method leading to 2,5-diheteroarylated thiophenes in good yields. We established that 2 mol % of air-stable PdCl(C_3_H_5_)(dppb) catalyst (and in some cases 0.5 mol % Pd(OAc)_2_ catalyst) in the presence of KOAc as the base promotes the 2,5-diheteroarylation of 2,5-dibromothiophene in the presence of a variety of heteroarenes such as thiophenes, furans, pyrroles, pyrazoles or isoxazoles as the coupling partners. The sequential diheteroarylation of 2,5-dibromothiophene was also found to be possible to afford 2,5-diheteroarylated thiophenes bearing two different heteroarene units. As both, 2,5-dibromothiophene and a wide variety of heteroarenes are commercially available, this method gives a convenient access to a large number of terthiophene derivatives.

## Supporting Information

File 1 Experimental procedures and characterization data.
