# Supplementary material for: Palladium-catalyzed 2,5-diheteroarylation of 2,5-dibromothiophene derivatives
Source: Beilstein J Org Chem. 2014 Dec 9;10:2912–9. doi: 10.3762/bjoc.10.309 (PMC4273267; doi:10.3762/bjoc.10.309)
Supplement: File 1 — Experimental procedures and characterization data. [file Beilstein_J_Org_Chem-10-2912-s001.pdf]

Supporting Information

for

Palladium-catalysed 2,5-diheteroarylation of 2,5-dibromothiophene derivatives

Fatma Belkessam[1,2], Aidene Mohand[2], Jean-François Soulé[1], Abdelhamid Elias[1,2*], Henri Doucet[1*]

Address: [1]Institut des Sciences Chimiques de Rennes, UMR 6226 CNRS-Université de Rennes, "Organométalliques: Matériaux et Catalyse", Campus de Beaulieu, 35042 Rennes, France, Tel.: 00-33-2-23-23-63-84, FAX 00-33-2-23-23-69-39 and [2]Département de chimie, Tizi Ouzou University, BP 17 RP 15000 Tizi-Ouzou, Algeria.

Email: Abdelhamid Elias - hamghil@yahoo.fr; Henri Doucet - henri.doucet@univ-rennes1.fr

*Corresponding author

Experimental procedures and characterization data

General information

All reactions were run under argon in Schlenk tubes using vacuum lines. DMA analytical grade was not distilled before use. KOAc (99%) was used. Commercial dibromothiophenes and heteroarenes were used without purification. The reactions were followed by GC and NMR. ^1H NMR and ^{13}C NMR spectra were recorded with a Bruker 400 MHz spectrometer in $CDCl_3$ solutions. Chemical shifts are reported in ppm relative to $CDCl_3$ (7.25 for ^1H NMR and 77.0 for ^{13}C NMR). Flash chromatography was performed on silica gel (230–400 mesh).

General procedure

In a typical experiment, the 2,5-dibromothiophene (1 mmol), heteroarene derivative (3 mmol), KOAc (0.294 g, 3 mmol) and $PdCl(C_3H_5)(dppb)$ (12.1 mg, 0.02 mmol), were dissolved in DMA (5 mL) under an argon atmosphere. The reaction mixture was stirred at 140 °C for 20h. After evaporation of the solvent, the product was purified by silica gel column chromatography.

Preparation of the $PdCl(C_3H_5)(dppb)$ catalyst [1]: An oven-dried 40 mL Schlenk tube equipped with a magnetic stirring bar under argon atmosphere, was charged with $[Pd(C_3H_5)Cl]_2$ (182 mg, 0.5 mmol) and dppb (426 mg, 1 mmol). 10 mL of anhydrous dichloromethane were added, then, the solution was stirred at room temperature for twenty minutes. The solvent was removed in vacuum. The yellow powder was used without purification. ^{31}P NMR (81 MHz, $CDCl_3$) δ = 19.3 (s).

5-(5-Bromothiophen-2-yl)-2-ethyl-4-methylthiazole (1a)

From 2,5-dibromothiophene (0.968 g, 4 mmol) and 2-ethyl-4-methylthiazole (0.127 g, 1 mmol) product **1a** was obtained in 52% (0.150 g) yield.

^1H NMR (400 MHz, $CDCl_3$) δ 6.98 (d, J = 3.8 Hz, 1H), 6.80 (d, J = 3.8 Hz, 1H), 2.95 (q, J = 7.6 Hz, 2H), 2.47 (s, 3H), 1.36 (t, J = 7.6 Hz, 3H).

δ ^{13}C NMR (100 MHz, $CDCl_3$) δ 170.2, 148.1, 135.3, 130.2, 126.6, 123.4, 112.1, 26.8, 16.3, 14.0.

$C_{10}H_{10}BrNS_2$ (288.23): Calcd C 41.67, H 3.50; Found C 41.80, H 3.38.

2,5-Bis(2-ethyl-4-methylthiazol-5-yl)thiophene (1b)

From 2,5-dibromothiophene (0.242 g, 1 mmol) and 2-ethyl-4-methylthiazole (0.381 g, 3 mmol) product **1b** was obtained in 81% (0.270 g) yield.

^1H NMR (400 MHz, $CDCl_3$) δ 6.96 (m, 2H), 2.94 (q, J = 7.6 Hz, 4H), 2.51 (s, 6H), 1.35 (t, J = 7.6 Hz, 6H).

δ ^{13}C NMR (100 MHz, $CDCl_3$) δ 169.9, 147.7, 133.9, 126.6, 123.9, 26.8, 16.5, 14.0.

$C_{16}H_{18}N_2S_3$ (334.52): Calcd C 57.45, H 5.42; Found C 57.30, H 5.28.

2,5-Bis(4-methylthiazol-5-yl)thiophene (2)

From 2,5-dibromothiophene (0.242 g, 1 mmol) and 4-methylthiazole (0.297 g, 3 mmol) product **2** was obtained in 82% (0.228 g) yield.

^1H NMR (400 MHz, $CDCl_3$) δ 8.58 (s, 2H), 7.10 (s, 2H), 2.64 (s, 6H).

^{13}C NMR (100 MHz, $CDCl_3$) δ 150.1, 149.6, 134.1, 127.5, 125.0, 16.6.

$C_{12}H_{10}N_2S_3$ (278.42): Calcd C 51.77, H 3.62; Found C 51.89, H 3.49.

5,5''-Dimethyl-2,2':5',2''-terthiophene (3) [2]

From 2,5-dibromothiophene (0.242 g, 1 mmol) and 2-methylthiophene (0.294 g, 3 mmol) product **3** was obtained in 74% (0.204 g) yield.

^1H NMR (400 MHz, $CDCl_3$) δ 6.24 (s, 2H), 6.21 (d, *J* = 3.6 Hz, 2H), 5.92 (d, *J* = 3.6 Hz, 2H), 1.62 (s, 6H).

^{13}C NMR (100 MHz, $CDCl_3$) δ 141.1, 137.7, 136.4, 128.2, 125.5, 125.4, 16.2.

5,5''-Dichloro-2,2':5',2''-terthiophene (4) [3]

From 2,5-dibromothiophene (0.242 g, 1 mmol) and 2-chlorothiophene (0.356 g, 3 mmol) product **3** was obtained in 77% (0.244 g) yield.

^1H NMR (400 MHz, $CDCl_3$) δ 6.97 (s, 2H), 6.91 (d, *J* = 3.8 Hz, 2H), 6.83 (d, *J* = 3.8 Hz, 2H).

^{13}C NMR (100 MHz, $CDCl_3$) δ 135.5, 135.4, 129.0, 126.9, 124.4, 122.9.

5,5''-Dicarbonitrile-2,2':5',2''-terthiophene (5) [4]

From 2,5-dibromothiophene (0.242 g, 1 mmol) and thiophene-2-carbonitrile (0.327 g, 3 mmol) product **3** was obtained in 62% (0.185 g) yield.

^1H NMR (400 MHz, $CDCl_3$) δ 7.55 (d, *J* = 3.6 Hz, 2H), 7.24 (s, 2H), 7.17 (d, *J* = 3.6 Hz, 2H).

^{13}C NMR (100 MHz, $CDCl_3$) δ 143.2, 138.3, 135.9, 126.9, 124.1, 113.8, 108.5.

5,5''-Diethyl [2,2':5',2''-Terthiophene]-5,5''-dicarboxylate (6)

From 2,5-dibromothiophene (0.242 g, 1 mmol) and ethyl thiophene-2-carboxylate (0.468 g, 3 mmol) product **6** was obtained in 58% (0.227 g) yield.

^1H NMR (400 MHz, $CDCl_3$) δ 7.68 (d, *J* = 3.9 Hz, 2H), 7.18 (s, 2H), 7.13 (d, *J* = 3.9 Hz, 2H), 4.35 (q, *J* = 7.6 Hz, 4H), 1.38 (t, *J* = 7.6 Hz, 6H).

^{13}C NMR (100 MHz, $CDCl_3$) δ 161.9, 143.0, 136.6, 134.0, 132.3, 125.9, 124.2, 61.3, 14.3.

$C_{18}H_{16}O_4S_3$ (392.51): Calcd C 55.08, H 4.11; Found C 55.01, H 4.24.

2,5-Di(2-thienyl)thiophene (7) [5]

From 2,5-dibromothiophene (0.242 g, 1 mmol) and thiophene (0.504 g, 6 mmol) product **7** was obtained in 85% (0.211 g) yield.

^{1}H NMR (400 MHz, $CDCl_3$) δ 7.22 (d, *J* = 5.1 Hz, 2H), 7.18 (d, *J* = 3.8 Hz, 2H), 7.09 (s, 2H), 7.03 (dd, *J* = 5.1, 3.8 Hz, 2H).

^{13}C NMR (100 MHz, $CDCl_3$) δ 137.0, 136.2, 127.8, 124.4, 124.3, 123.7.

2,5-Bis(5-butylfuran-2-yl)thiophene (8)

From 2,5-dibromothiophene (0.242 g, 1 mmol) and 2-*n*-butylfuran (0.372 g, 3 mmol) product **8** was obtained in 79% (0.259 g) yield.

^{1}H NMR (400 MHz, $CDCl_3$) δ 7.09 (s, 2H), 6.39 (d, *J* = 3.1 Hz, 2H), 6.04 (d, *J* = 3.1 Hz, 2H), 2.68 (t, *J* = 7.6 Hz, 4H), 1.68 (quint., *J* = 7.6 Hz, 4H), 1.43 (sext., *J* = 7.6 Hz, 4H), 0.97 (t, *J* = 7.6 Hz, 6H).

^{13}C NMR (100 MHz, $CDCl_3$) δ 156.1, 147.5, 132.0, 122.0, 106.9, 105.7, 30.2, 27.8, 22.2, 13.8.

$C_{20}H_{24}O_2S$ (328.47): Calcd C 73.13, H 7.36; Found C 73.21, H 7.24.

1,1'-(5,5'-(Thiophene-2,5-diyl)bis(furan-5,2-diyl))diethanone (9)

From 2,5-dibromothiophene (0.242 g, 1 mmol) and 2-acetylfuran (0.330 g, 3 mmol) product **9** was obtained in 60% (0.180 g) yield.

^{1}H NMR (400 MHz, $CDCl_3$) δ 7.43 (s, 2H), 7.24 (d, *J* = 3.6 Hz, 2H), 6.67 (d, *J* = 3.6 Hz, 2H), 2.52 (s, 6H).

^{13}C NMR (100 MHz, $CDCl_3$) δ 186.0, 152.1, 151.7, 132.8, 126.1, 119.4, 108.2, 26.0.

$C_{16}H_{12}O_4S$ (300.33): Calcd C 63.99, H 4.03; Found C 63.87, H 4.00.

Dimethyl 5,5'-(thiophene-2,5-diyl)bis(2-methylfuran-3-carboxylate) (10)

From 2,5-dibromothiophene (0.242 g, 1 mmol) and methyl 2-methylfuran-3-carboxylate (0.420 g, 3 mmol) product **10** was obtained in 63% (0.227 g) yield.

^{1}H NMR (400 MHz, $CDCl_3$) δ 7.14 (s, 2H), 6.72 (s, 2H), 3.84 (s, 6H), 2.63 (s, 6H).

^{13}C NMR (100 MHz, $CDCl_3$) δ 164.1, 158.6, 146.8, 131.5, 123.3, 115.2, 105.6, 51.4, 13.8.

$C_{18}H_{16}O_6S$ (360.38): Calcd C 59.99, H 4.47; Found C 60.20, H 4.21.

2,5-Bis(1-methylpyrrol-2-yl)thiophene (11) [6]

From 2,5-dibromothiophene (0.242 g, 1 mmol) and 1-methylpyrrole (0.405 g, 5 mmol) product **11** was obtained in 78% (0.189 g) yield.

^{1}H NMR (400 MHz, $CDCl_3$) δ 6.97 (s, 2H), 6.72 (m, 2H), 6.36 (dd, *J* = 3.0, 1.4 Hz, 2H), 6.19 (t, *J* = 3.0 Hz, 2H), 3.77 (s, 6H).

^{13}C NMR (100 MHz, $CDCl_3$) δ 133.9, 126.9, 124.9, 124.1, 109.8, 107.9, 35.2.

2,5-Bis(3,5-dimethylisoxazol-4-yl)thiophene (12)

From 2,5-dibromothiophene (0.242 g, 1 mmol) and 3,5-dimethylisoxazole (0.291 g, 3 mmol) product **12** was obtained in 80% (0.219 g) yield.

^{1}H NMR (400 MHz, $CDCl_3$) δ 6.99 (s, 2H), 2.51 (s, 6H), 2.36 (s, 6H).

^{13}C NMR (100 MHz, $CDCl_3$) δ 165.8, 158.3, 131.3, 126.4, 110.2, 12.0, 11.1.

$C_{14}H_{14}N_2O_2S$ (274.34): Calcd C 61.29, H 5.14; Found C 61.17, H 5.05.

2,5-Bis(5-chloro-1,3-dimethylpyrazole-4-yl) thiophene (13)

From 2,5-dibromothiophene (0.242 g, 1 mmol) and 5-chloro-1,3-dimethylpyrazole (0.392 g, 3 mmol) product **13** was obtained in 83% (0.283 g) yield.

^{1}H NMR (400 MHz, $CDCl_3$) δ 7.09 (s, 2H), 3.77 (s, 6H), 2.36 (s, 6H).

^{13}C NMR (100 MHz, $CDCl_3$) δ 146.0, 131.8, 125.0, 111.1, 36.0, 13.9.

$C_{14}H_{14}Cl_2N_4S$ (341.26): Calcd C 49.27, H 4.14; Found C 49.40, H 4.27.

5,5'-(3-Methylthiophene-2,5-diyl)bis(2-ethyl-4-methylthiazole) (14)

From 2,5-dibromo-3-methylthiophene (0.256 g, 1 mmol) and 2-ethyl-4-methylthiazole (0.381 g, 3 mmol) product **14** was obtained in 83% (0.289 g) yield.

^{1}H NMR (400 MHz, $CDCl_3$) δ 6.87 (s, 1H), 2.84-2.74 (m, 4H), 2.32 (s, 3H), 2.14 (s, 3H), 2.00 (s, 3H), 1.23-1.13 (m, 6H).

^{13}C NMR (100 MHz, $CDCl_3$) δ 172.8, 170.6, 152.0, 149.3, 139.3, 135.2, 131.0, 128.4, 125.5, 123.0, 28.1, 28.0, 17.7, 17.1, 15.6, 15.0, 14.9.

$C_{17}H_{20}N_2S_3$ (348.55): Calcd C 58.58, H 5.78; Found C 58.41, H 5.58.

5,5'-(3-Methylthiophene-2,5-diyl)bis(4-methylthiazole) (15)

From 2,5-dibromo-3-methylthiophene (0.256 g, 1 mmol) and 4-methylthiazole (0.297 g, 3 mmol) product **15** was obtained in 80% (0.234 g) yield.

^{1}H NMR (400 MHz, $CDCl_3$) δ 8.75 (s, 1H), 8.60 (s, 1H), 6.97 (s, 1H), 2.61 (s, 3H), 2.43 (s, 3H), 2.16 (s, 3H).

^{13}C NMR (100 MHz, $CDCl_3$) δ 152.1, 151.2, 149.9, 149.1, 138.0, 133.4, 129.8, 126.8, 125.1, 122.8, 16.6, 15.9, 14.6.

$C_{13}H_{12}N_2S_3$ (292.44): Calcd C 53.39, H 4.14; Found C 53.29, H 4.27.

3',5,5''-Trimethyl-2,2':5',2''-terthiophene (16) [7]

From 2,5-dibromo-3-methylthiophene (0.256 g, 1 mmol) and 2-methylthiophene (0.294 g, 3 mmol) product **16** was obtained in 73% (0.212 g) yield.

^{1}H NMR (400 MHz, $CDCl_3$) δ 6.95-6.80 (m, 3H), 6.75 (s, 1H), 6.70 (s, 1H), 2.53 (s, 6H), 2.36 (s, 3H).

^{13}C NMR (100 MHz, AcetoneD$_6$) δ 141.3, 140.7, 136.1, 136.0, 135.6, 135.3, 131.1, 128.8, 127.9, 127.6, 127.0, 125.1, 16.2, 15.9, 15.8.

5,5''-Dichloro-3'-methyl-2,2':5',2''-terthiophene (17)

From 2,5-dibromo-3-methylthiophene (0.256 g, 1 mmol) and 2-chlorothiophene (0.356 g, 3 mmol) product **17** was obtained in 74% (0.245 g) yield.

^{1}H NMR (400 MHz, $CDCl_3$) δ 7.07 (d, J = 3.8 Hz, 1H), 7.07 (s, 1H), 7.03 (s, 2H), 6.98 (d, J = 3.8 Hz, 1H), 2.31 (s, 3H).

^{13}C NMR (100 MHz, AcetoneD$_6$) δ 137.3, 137.1, 136.2, 135.5, 130.7, 130.5, 130.1, 129.7, 129.4, 129.0, 127.1, 125.1, 16.2.

$C_{13}H_8Cl_2S_3$ (331.30): Calcd C 47.13, H 2.43; Found C 47.11, H 2.34.

5,5''-Diethyl [3'-methyl-2,2':5',2''-terthiophene]-5,5''-dicarboxylate (18)

From 2,5-dibromo-3-methylthiophene (0.256 g, 1 mmol) and ethyl thiophene-2-carboxylate (0.468 g, 3 mmol) product **18** was obtained in 60% (0.244 g) yield.

^{1}H NMR (400 MHz, $CDCl_3$) δ 7.87 (d, J = 3.9 Hz, 1H), 7.81 (d, J = 3.9 Hz, 1H), 7.30-7.25 (m, 2H), 7.21 (s, 1H), 4.55-4.45 (m, 4H), 2.55 (s, 3H), 1.55-1.40 (m, 6H).

^{13}C NMR (100 MHz, $CDCl_3$) δ 162.0, 161.9, 143.1, 142.6, 136.4, 134.7, 134.0, 133.6, 132.9, 132.2, 130.9, 129.5, 125.6, 124.0, 61.2, 15.8, 14.4, 14.3.

$C_{19}H_{18}O_4S_3$ (406.54): Calcd C 56.13, H 4.46; Found C 55.98, H 4.57.

3'-Methyl-2,2':5',2''-terthiophene (19) [8]

From 2,5-dibromo-3-methylthiophene (0.256 g, 1 mmol) and thiophene (0.504 g, 6 mmol) product **19** was obtained in 78% (0.204 g) yield.

^{1}H NMR (400 MHz, $CDCl_3$) δ 7.46 (d, J = 4.8 Hz, 1H), 7.37 (d, J = 4.8 Hz, 1H), 7.25-7.20 (m, 2H), 7.10-7.02 (m, 3H), 2.33 (s, 3H).

Dimethyl 5,5'-(3-methylthiophene-2,5-diyl)bis(2-methylfuran-3-carboxylate) (20)

From 2,5-dibromo-3-methylthiophene (0.256 g, 1 mmol) and methyl 2-methylfuran-3-carboxylate (0.420 g, 3 mmol) product **20** was obtained in 51% (0.191 g) yield.

^{1}H NMR (400 MHz, $CDCl_3$) δ 7.00 (s, 1H), 6.70 (s, 1H), 6.65 (s, 1H), 3.85 (s, 3H), 3.84 (s, 3H), 2.63 (s, 3H), 2.62 (s, 3H), 2.35 (s, 3H).

^{13}C NMR (100 MHz, $CDCl_3$) δ 164.4, 164.1, 156.5, 156.1, 147.0, 146.9, 134.4, 130.0, 126.7, 125.7, 115.2, 115.1, 106.8, 105.5, 54.0, 51.4, 15.6, 13.8, 13.7.

$C_{19}H_{18}O_6S$ (374.41): Calcd C 60.95, H 4.85; Found C 60.99, H 4.78.

2,2'-(3-Methylthiophene-2,5-diyl)bis(1-methylpyrrole) (21)

From 2,5-dibromo-3-methylthiophene (0.256 g, 1 mmol) and 1-methylpyrrole (0.405 g, 5 mmol)) product **21** was obtained in 77% (0.197 g) yield.

^{1}H NMR (400 MHz, $CDCl_3$) δ 6.86 (s, 1H), 6.75 (m, 1H), 6.70 (m, 1H), 6.34 (dd, J = 3.6, 1.8 Hz, 1H), 6.24 (dd, J = 3.6, 1.8 Hz, 1H), 6.21 (t, J = 3.4 Hz, 1H), 6.17 (t, J = 3.4 Hz, 1H), 3.77 (s, 3H), 3.59 (s, 3H), 2.18 (s, 3H).

^{13}C NMR (100 MHz, $CDCl_3$) δ 136.8, 133.9, 127.4, 127.3, 127.1, 125.3, 124.1, 123.2, 111.2, 109.7, 108.0, 107.5, 35.3, 34.5, 14.9.

$C_{15}H_{16}N_2S$ (256.37): Calcd C 70.27, H 6.29; Found C 70.37, H 6.37.

2,5-Bis(3,5-dimethylisoxazol-4-yl)-3-methylthiophene (22)

From 2,5-dibromo-3-methylthiophene (0.256 g, 1 mmol) and 3,5-dimethylisoxazole (0.291 g, 3 mmol) product **22** was obtained in 75% (0.216 g) yield.

^{1}H NMR (400 MHz, $CDCl_3$) δ 6.86 (s, 1H), 2.52 (s, 3H), 2.38 (s, 3H), 2.35 (s, 3H), 2.22 (s, 3H), 2.10 (s, 3H).

^{13}C NMR (100 MHz, $CDCl_3$) δ 167.3, 165.6, 159.8, 158.4, 137.2, 131.3, 128.8, 124.4, 110.3, 109.1, 14.4, 12.1, 11.7, 11.3, 10.6.

$C_{15}H_{16}N_2O_2S$ (288.36): Calcd C 62.48, H 5.59; Found C 62.19, H 5.69.

2-Ethyl-5-(5-(2-isobutylthiazol-5-yl)thiophen-2-yl)-4-methylthiazole (23)

From 5-(5-bromothiophen-2-yl)-2-ethyl-4-methylthiazole **1a** (0.288 g, 1 mmol) and 2-isobutylthiazole (0.211 g, 1.5 mmol) product **23** was obtained in 89% (0.310 g) yield.

^{1}H NMR (400 MHz, $CDCl_3$) δ 7.71 (s, 1H), 7.05 (d, J = 3.8 Hz, 1H), 6.97 (d, J = 3.8 Hz, 1H), 2.97 (q, J = 7.6 Hz, 2H), 2.85 (d, J = 7.6 Hz, 2H), 2.54 (s, 3H), 2.18-2.07 (m, 1H), 1.38 (t, J = 7.6 Hz, 3H), 1.01 (d, J = 7.6 Hz, 6H).

^{13}C NMR (100 MHz, $CDCl_3$) δ 170.0, 169.4, 147.9, 137.9, 133.5, 133.4, 131.1, 126.9, 125.5, 123.9, 42.3, 29.7, 26.8, 22.2, 16.6, 14.0.

$C_{17}H_{20}N_2S_3$ (348.55): Calcd C 58.58, H 5.78; Found C 58.40, H 5.71.

2-Ethyl-4-methyl-5-(5'-methyl-2,2'-bithiophen-5-yl)thiazole (24)

From 5-(5-bromothiophen-2-yl)-2-ethyl-4-methylthiazole **1a** (0.288 g, 1 mmol) and 2-methylthiophene (0.147 g, 1.5 mmol) product **24** was obtained in 70% (0.213 g) yield.

^{1}H NMR (400 MHz, $CDCl_3$) δ 7.01 (d, J = 3.8 Hz, 1H), 6.96 (d, J = 3.8 Hz, 1H), 6.94 (d, J = 3.8 Hz, 1H), 6.66 (d, J = 3.8 Hz, 1H), 2.97 (q, J = 7.6 Hz, 2H), 2.55 (s, 3H), 2.48 (s, 3H), 1.39 (t, J = 7.6 Hz, 3H).

^{13}C NMR (100 MHz, $CDCl_3$) δ 169.8, 147.6, 139.5, 138.0, 134.5, 132.0, 127.0, 126.0, 124.4, 123.7, 123.2, 26.9, 16.6, 15.3, 14.1.

$C_{15}H_{15}NS_3$ (305.48): Calcd C 58.98, H 4.95; Found C 59.04, H 5.09.

5'-(2-Ethyl-4-methylthiazol-5-yl)-2,2'-bithiophene-5-carbonitrile (25)

From 5-(5-bromothiophen-2-yl)-2-ethyl-4-methylthiazole **1a** (0.288 g, 1 mmol) and thiophene-2-carbonitrile (0.164 g, 1.5 mmol) product **25** was obtained in 67% (0.212 g) yield.

^{1}H NMR (400 MHz, $CDCl_3$) δ 7.52 (d, *J* = 3.8 Hz, 1H), 7.22 (d, *J* = 3.8 Hz, 1H), 7.12 (d, *J* = 3.8 Hz, 1H), 7.01 (d, *J* = 3.8 Hz, 1H), 2.99 (q, *J* = 7.6 Hz, 2H), 2.56 (s, 3H), 1.39 (t, *J* = 7.6 Hz, 3H).

^{13}C NMR (100 MHz, $CDCl_3$) δ 170.6, 148.4, 143.9, 138.1, 135.4, 134.4, 127.2, 126.2, 123.5, 123.2, 113.9, 107.4, 26.8, 16.6, 13.9.

$C_{15}H_{12}N_2S_3$ (316.46): Calcd C 56.93, H 3.82; Found C 57.10, H 3.99.

5-(2,2'-Bithiophen-5-yl)-2-ethyl-4-methylthiazole (26)

From 5-(5-bromothiophen-2-yl)-2-ethyl-4-methylthiazole **1a** (0.288 g, 1 mmol) and thiophene (0.202 g, 2 mmol) product **26** was obtained in 41% (0.119 g) yield.

^{1}H NMR (400 MHz, $CDCl_3$) δ 7.23 (d, *J* = 5.1 Hz, 1H), 7.18 (d, *J* = 3.8 Hz, 1H), 7.11 (d, *J* = 3.8 Hz, 1H), 7.03 (dd, *J* = 5.1, 3.8 Hz, 1H), 6.98 (d, *J* = 3.8 Hz, 1H), 3.01 (q, *J* = 7.6 Hz, 2H), 2.58 (s, 3H), 1.40 (t, *J* = 7.6 Hz, 3H).

^{13}C NMR (100 MHz, $CDCl_3$) δ 147.6, 137.6, 136.8, 132.5, 127.9, 127.2, 124.7, 124.4, 124.0, 123.8, 26.8, 16.6, 14.2.

$C_{14}H_{13}NS_3$ (291.45): Calcd C 57.69, H 4.50; Found C 57.47, H 4.71.

2-ethyl-4-methyl-5-(5-(1-methylpyrrol-2-yl)thiophen-2-yl)thiazole (27)

From 5-(5-bromothiophen-2-yl)-2-ethyl-4-methylthiazole **1a** (0.288 g, 1 mmol) and 1-methylpyrrole (0.162 g, 2 mmol) product **27** was obtained in 74% (0.213 g) yield.

^{1}H NMR (400 MHz, $CDCl_3$) δ 7.02 (d, *J* = 3.8 Hz, 1H), 6.96 (d, *J* = 3.8 Hz, 1H), 6.73-6.70 (m, 1H), 6.36 (dd, *J* = 3.0, 1.4 Hz, 1H), 6.17 (t, *J* = 3.0 Hz, 1H), 3.75 (s, 3H), 2.98 (q, *J* = 7.6 Hz, 2H), 2.57 (s, 3H), 1.40 (t, *J* = 7.6 Hz, 3H).

^{13}C NMR (100 MHz, $CDCl_3$) δ 169.6, 147.3, 135.1, 132.4, 126.5, 126.4, 124.8, 124.3, 124.2, 110.0, 107.9, 35.1, 26.7, 16.4, 14.0.

$C_{15}H_{16}N_2S_2$ (288.43): Calcd C 62.46, H 5.59; Found C 62.57, H 5.67.

Methyl 5-(5-(2-ethyl-4-methylthiazol-5-yl)thiophen-2-yl)-2-methylfuran-3-carboxylate (28)

From 5-(5-bromothiophen-2-yl)-2-ethyl-4-methylthiazole **1a** (0.288 g, 1 mmol) and methyl 2-methylfuran-3-carboxylate (0.210 g, 1.5 mmol) product **28** was obtained in 62% (0.215 g) yield.

^{1}H NMR (400 MHz, $CDCl_3$) δ 7.15 (d, *J* = 3.8 Hz, 1H), 6.98 (d, *J* = 3.8 Hz, 1H), 6.73 (s, 1H), 3.84 (s, 3H), 2.98 (q, *J* = 7.6 Hz, 2H), 2.63 (s, 3H), 2.55 (s, 3H), 1.38 (t, *J* = 7.6 Hz, 3H).

^{13}C NMR (100 MHz, $CDCl_3$) δ 170.1, 164.1, 158.6, 147.8, 146.7, 132.9, 132.6, 126.9, 124.2,

123.2, 115.2, 105.6, 51.4, 26.8, 16.6, 14.1, 13.8.

$C_{17}H_{17}NO_3S_2$ (347.45): Calcd C 58.77, H 4.93; Found C 58.97, H 4.69.

4-(5-(2-Ethyl-4-methylthiazol-5-yl)thiophen-2-yl)-3,5-dimethylisoxazole (29)

From 5-(5-bromothiophen-2-yl)-2-ethyl-4-methylthiazole **1a** (0.288 g, 1 mmol) and 3,5-dimethylisoxazole (0.146 g, 1.5 mmol) product **29** was obtained in 66% (0.201 g) yield.

^{1}H NMR (400 MHz, $CDCl_3$) δ 7.06 (d, J = 3.8 Hz, 1H), 6.94 (d, J = 3.8 Hz, 1H), 2.98 (q, J = 7.6 Hz, 2H), 2.55 (s, 3H), 2.52 (s, 3H), 2.37 (s, 3H), 1.39 (t, J = 7.6 Hz, 3H).

^{13}C NMR (100 MHz, $CDCl_3$) δ 170.2, 165.9, 158.4, 147.9, 133.9, 131.5, 126.7, 126.5, 123.9, 110.2, 26.9, 16.6, 14.1, 12.1, 11.2.

$C_{15}H_{16}N_2OS_2$ (304.43): Calcd C 59.18, H 5.30; Found C 59.07, H 5.22.

5-(5-(5-Chloro-1,3-dimethylpyrazol-4-yl)thiophen-2-yl)-2-ethyl-4-methylthiazole (30)

From 5-(5-bromothiophen-2-yl)-2-ethyl-4-methylthiazole **1a** (0.288 g, 1 mmol) and 5-chloro-1,3-dimethylpyrazole (0.196 g, 1.5 mmol) product **30** was obtained in 72% (0.243 g) yield.

^{1}H NMR (400 MHz, $CDCl_3$) δ 7.10 (d, J = 3.8 Hz, 1H), 7.04 (d, J = 3.8 Hz, 1H), 3.82 (s, 3H), 2.98 (q, J = 7.6 Hz, 2H), 2.56 (s, 3H), 2.39 (s, 3H), 1.39 (t, J = 7.6 Hz, 3H).

^{13}C NMR (100 MHz, $CDCl_3$) δ 169.9, 147.5, 146.1, 133.3, 132.7, 126.5, 125.5, 125.3, 124.3, 111.0, 36.2, 26.9, 16.6, 14.2, 14.0.

$C_{15}H_{16}ClN_3S_2$ (337.89): Calcd C 53.32, H 4.77; Found C 53.50, H 4.87.

5-Bromo-5'-methyl-2,2'-bithiophene (31) [9]

From 2,5-dibromothiophene (0.242 g, 1 mmol) and 2-methylthiophene (0.294 g, 3 mmol) product **31** was obtained in 35% (0.090 g) yield.

^{1}H NMR (400 MHz, $CDCl_3$) δ 6.82 (d, J = 3.8 Hz, 1H), 6.78 (d, J = 3.8 Hz, 1H), 6.70 (d, J = 3.8 Hz, 1H), 6.54 (d, J = 3.8 Hz, 1H), 2.36 (s, 3H).

2,2'-Bithiophene (32)

From 2-bromothiophene (0.163 g, 1 mmol) and thiophene (0.504 g, 6 mmol) product **32** was obtained in 58% (0.096 g) yield.

^{1}H NMR (400 MHz, $CDCl_3$) δ 7.19 (d, J = 5.1 Hz, 1H), 7.17 (d, J = 3.8 Hz, 1H),7.00 (dd, J = 5.1, 3.8 Hz, 1H).

1-Methyl-2-(thiophen-2-yl)-pyrrole (33) [10]

From 2-bromothiophene (0.163 g, 1 mmol) and 1-methylpyrrole (0.486 g, 6 mmol) product **33** was obtained in 61% (0.099 g) yield.

^{1}H NMR (400 MHz, $CDCl_3$) δ 7.25 (d, J = 5.1 Hz, 1H), 7.06 (dd, J = 5.1, 3.8 Hz, 1H), 7.02 (d, J

= 3.8 Hz, 1H), 6.72-6.68 (m, 1H), 6.35-6.30 (m, 1H), 6.16 (t, *J* = 4.0 Hz, 1H), 3.71 (s, 3H).

4,7-Bis(2-ethyl-4-methylthiazol-5 yl)benzo[c][1,2,5]thiadiazole (34)

From 4,7-dibromobenzo[c][1,2,5]thiadiazole (0.294 g, 1 mmol) and 2-ethyl-4-methylthiazole (0.381 g, 3 mmol) product **34** was obtained in 92% (0.355 g) yield.

^{1}H NMR (400 MHz, $CDCl_3$) δ 7.66 (s, 2H), 3.05 (q, *J* = 7.6 Hz, 4H), 2.52 (s, 6H), 1.42 (t, *J* = 7.6 Hz, 6H).

^{13}C NMR (100 MHz, $CDCl_3$) δ 172.5, 153.6, 150.1, 129.1, 125.3, 125.2, 26.8, 16.9, 14.0.

$C_{18}H_{18}N_4S_3$ (386.56): Calcd C 55.93, H 4.69; Found C 55.78, H 4.54.

4,7-Bis(5-methylthiophen-2-yl)benzo[c][1,2,5]thiadiazole (35) [11]

From 4,7-dibromobenzo[c][1,2,5]thiadiazole (0.294 g, 1 mmol) and 2-methylthiophene (0.294 g, 3 mmol) product **35** was obtained in 86% (0.282 g) yield.

^{1}H NMR (400 MHz, $CDCl_3$) δ 7.75 (d, *J* = 3.6 Hz, 2H), 7.75 (s, 2H), 6.85 (d, *J* = 3.6 Hz, 2H), 2.57 (s, 6H).

^{13}C NMR (100 MHz, $CDCl_3$) δ 152.6, 141.5, 137.1, 127.5, 126.3, 125.7, 125.1, 15.4.

4,7-Di(thiophen-2-yl)benzo[c][1,2,5]thiadiazole (36) [12]

From 4,7-dibromobenzo[c][1,2,5]thiadiazole (0.294 g, 1 mmol) and thiophene (0.504 g, 6 mmol) product **36** was obtained in 82% (0.246 g) yield.

^{1}H NMR (400 MHz, CD_2Cl_2) δ 8.12-8.07 (m, 2H), 7.88-7.79 (m, 2H), 7.47 (t, *J* = 4.1 Hz, 2H), 7.20 (dd, *J* = 7.0, 4.3 Hz, 2H).

^{13}C NMR (100 MHz, CD_2Cl_2) δ 153.1, 139.9, 128.4, 128.0, 127.4, 126.3, 126.1.

4,7-Bis(1-methylpyrrol-2-yl)benzo[c][1,2,5]thiadiazole (37) [13]

From 4,7-dibromobenzo[c][1,2,5]thiadiazole (0.294 g, 1 mmol) and 1-methylpyrrole (0.405 g, 5 mmol)) product **37** was obtained in 78% (0.229 g) yield.

^{1}H NMR (400 MHz, $CDCl_3$) δ 7.58 (s, 2H), 6.87 (m, 2H), 6.57 (dd, *J* = 3.6, 1.8 Hz, 2H), 6.33 (t, *J* = 3.0 Hz, 2H), 3.71 (s, 6H).

^{13}C NMR (100 MHz, $CDCl_3$) δ 154.3, 130.1, 128.7, 125.4, 124.9, 111.9, 108.4, 35.7.

4,7-Bis(3,5-dimethylisoxazol-4-yl)benzo[c][1,2,5]thiadiazole (38)

From 4,7-dibromobenzo[c][1,2,5]thiadiazole (0.294 g, 1 mmol) and 3,5-dimethylisoxazole (0.291 g, 3 mmol) product **38** was obtained in 85% (0.277 g) yield.

^{1}H NMR (400 MHz, $CDCl_3$) δ 7.53 (s, 2H), 2.43 (s, 6H), 2.29 (s, 6H).

^{13}C NMR (100 MHz, $CDCl_3$) δ 167.1, 159.1, 153.9, 129.7, 123.8, 112.8, 12.1, 11.1.

$C_{16}H_{14}N_4O_2S$ (326.37): Calcd C 58.88, H 4.32; Found C 58.97, H 4.20.

References

[1] Cantat, T.; Génin, E.; Giroud, C.; Meyer G.; Jutand, A. *J. Organomet. Chem.* 2003, **687**, 365-376.

[2] Beny, J. P.; Dhawan, S. N.; Kagan, J.; Sundlass, S. *J. Org. Chem.* **1982**, *47*, 2201-2204.

[3] Kim, E. J.; Jung, K.-J.; Lee, I.-H.; Kim, B. R.; Kim, J.-J.; Park, J. K.; Lee, S.-G.; Yoon, Y.-J. Bull. *Korean Chem. Soc.* **2010**, *31*, 2985-2988.

[4] Hu, J.; Liang, L.; Chen, T.; Liu, P.; Deng, W. *Dyes Pigments* **2014**, 158-161.

[5] Hassan Omar, O.; Babudri, F.; Farinola, G. M.; Naso, F.; Operamolla, A.; Pedone, A. *Tetrahedron* **2011**, *67*, 486-494.

[6] Borgwarth, K.; Rohde, N.; Ricken, C.; Hallensleben, M. L.; Mandler, D.; Heinze, J. *Adv. Mater.* **1999**, *11*, 1221-1226.

[7] Kriste, B.; Tian, P.; Kossmehl, G.; Engelmann, G.; Jugelt, W. *Magn. Reson. Chem.* **1995**, *33*, 70-76.

[8] Jadamiec, M.; Lapkowski, M.; Matlengiewicz, M.; Brembilla, A.; Henry, B.; Rodehueser, L. *Electrochim. Acta* **2007**, *52*, 6146-6154.

[9] Nagaki, A.; Takabayashi, N.; Tomida, Y.; Yoshida, J.-i. *Org. Lett.* **2008**, *10*, 3937-3940.

[10] Lotz, S.; Crause, C.; Olivier, A. J.; Liles, D. C.; Goerls, H.; Landman, M.; Bezuidenhout, D. I. *Dalton Trans.* **2009**, 697-710.

[11] Chen, M.; Haeussler, M.; Moad, G.; Rizzardo, E. *Org. Biomol. Chem.* **2011**, *9*, 6111-6119.

[12] Kim, J.-J.; Choi, H.; Lee, J.-W.; Kang, M.-S.; Song, K.; Kang, S. O.; Ko, J. *J. Mater. Chem.* **2008**, *18*, 5223-5229.

[13] Sun, M.; Lan, L.; Wang, L.; Peng, J.; Cao, Y. *Macromol. Chem. Physics* **2008**, *209*, 2504-2509.
